# Development and Characterization of a Hand Rub Gel Produced with Artisan Alcohol (*Puntas*), Silver Nanoparticles, and Saponins from Quinoa

**DOI:** 10.3390/gels10040234

**Published:** 2024-03-29

**Authors:** Oscar Analuiza, Belen Paredes, Alejandra Lascano, Santiago Bonilla, José-Luis Martínez-Guitarte

**Affiliations:** 1 International School of Doctorate (EIDUNED), National University of Distance Education (UNED), 28040 Madrid, Spain; jlmartinez@ccia.uned.es; 2 Faculty of Civil and Mechanical Engineering, Technical University of Ambato, Ambato 180104, Ecuador; mb.paredes@uta.edu.ec (B.P.); am.lascano@uta.edu.ec (A.L.); 3 Independent Researcher, Ambato 180215, Ecuador; santiago85bonilla@gmail.com

**Keywords:** pandemic, sanitizers, artisan alcohol, silver nanoparticles, saponins

## Abstract

The emergence of the global pandemic (COVID-19) has directed global attention towards the importance of hygiene as the primary defense against various infections. In this sense, one of the frequent recommendations of the World Health Organization (WHO) is regular hand washing and the use of alcohol-based hand sanitizers. Ethanol is the most widely used alcohol due to its effectiveness in eliminating pathogens, ease of use, and widespread production. However, artisanal alcohol, generally used as a spirit drink, could be a viable alternative for developing sanitizing gels. In this study, the use of alcohol “*Puntas*”, silver nanoparticles, and saponins from quinoa was evaluated to produce hand sanitizer gels. The rheological, physicochemical, and antimicrobial properties were evaluated. In the previous assays, the formulations were adjusted to be similar in visual viscosity to the control gel. A clear decrease in the apparent viscosity was observed with increasing shear rate, and an inversely proportional relationship was observed with the amount of ethyl alcohol used in the formulations. The flow behavior index (n) values reflected a pseudoplastic behavior. Oscillatory dynamic tests were performed to analyze the viscoelastic behavior of gels. A decrease in storage modulus (G′) and an increase in loss modulus (G″) as a function of the angular velocity (ω) was observed. The evaluation of pH showed that the gels complied with the requirements to be in contact with the skin of the people, and the textural parameters showed that the control gel was the hardest. The use of artisan alcohol could be an excellent alternative to produce sanitizer gel and contribute to the requirements of the population.

## 1. Introduction

The COVID-19 pandemic generated several requirements for healthcare people; using antibacterial gels for application on the hands was one of the most significant items that healthcare industries generated [1,2]. In this sense, effective materials to prevent the spread of diseases have been evaluated [3,4]. Nano-structured materials have been used recurrently [5]. Silver nanoparticles are one of the most used nano-structured materials and have gained popularity due to their excellent bioactive properties [5,6]. Silver nanoparticles represent a significant advancement in nanotechnology, with their distinct physicochemical properties and potent antimicrobial characteristics rendering them suitable for a wide array of applications [7,8]. The production of antibacterial products has been focused on new materials with a reduced effect on environmental contamination; the use of materials from agricultural waste [6,9], phytochemicals from plants [10,11], anti-nutritional components of food [12,13], and microalgae from wastewater [14,15] has been evaluated. The use of antimicrobial materials from more environmentally friendly sources as functional agents for cosmetic and medical products is based on the high antimicrobial activities and their relative eco-compatibility [16,17]. Gels are semi-solid pharmaceutical forms formed by a solvent thickened by the addition of substances of a colloidal nature [18,19]. These colloids are gelling polymers that constitute the dispersed phase, and the liquid solvent is the phase that keeps going [20,21]. The most common occurrence is that the continuous phase comprises water or hydroalcoholic solutions (hydrogels) [22]. The World Health Organization mentions that a sanitizing hand rub is “An alcohol-containing preparation (liquid, gel or foam) designed for application to the hands to inactivate microorganisms and temporarily suppress their growth [23]. Such preparations may contain one or more types of alcohol, other active ingredients with excipients, and humectants” [24,25,26].

The most used alcohol for gel development is medicinal; however, the use of other alcohol types could be a suitable variant [27]; in this sense, ethyl alcohol, which in many countries is produced using an artisan method named “*trapiche*”, could be used for developing new and effective products [28,29]. The alcohol produced by the trapiches method is named “*puntas*”, which is a traditional name for this type of alcohol in Ecuador, whereas in Colombia, it is called “*aguardiente*” [30,31] or “*cachaza*” in Brazil [32,33]. Ethyl alcohol is destined for human consumption; however, the procedure that is used may not be safe for human consumption [34,35]. Sanitary organisms always seize this beverage and destroy it by incineration or throwing it into the sewers, producing contamination and other collateral effects. In many countries, bioethanol is produced as an ingredient for beauty and cosmetics products, pharmaceuticals, beverages, food, and as an oxygenated additive of gasoline [36,37]. Based on the requirements for health care, the use of this type of alcohol will be a viable alternative in different pharmaceutical applications, such as hydrogels or creams for sanitization [24,38].

On the other hand, researches refer to skin toxicity due to high alcohol content [39,40]; in this sense, the use of materials to increase microbiological activity will be the best strategy to reduce the amount of alcohol [2,41]. Some plant extracts have been used to increase microbial activity; for example, a gel developed with extract of coriander (*Coriandrum Sativum* L.) seeds against *Staphylococcus Aureus* showed a moderate inhibition category [1]; formulations prepared from natural ingredients that include Aloe vera, vitamin E, glycerin, and different essential oils showed an excellent effect on Gram-positive and Gram-negative bacteria, as well as an opportunistic pathogenic yeast (*C. albicans*) [42]; and the use of *Calendula officinalis* and Aloe vera was well tolerated by the skin, increasing the hydration of the stratum corneum [43]. Using waste material from agricultural or food production is a good strategy; in this sense, the saponins prevenient from quinoa preparation are a viable alternative. The quinoa saponins showed different anti-bactericidal effects against six types of bacteria: *Staphylococcus aureus*, *Staphylococcus epidermidis*, *Bacillus cereus*, *Salmonella enteritidis*, *Pseudomonas aeruginosa*, and *Listeria ivanovii* [44]. In this study, ethyl alcohol (artisan alcohol, “*puntas*”) with silver nanoparticles and quinoa saponins were mixed to produce a sanitizing hand rub gel; the rheological, physical, and antimicrobial properties of the gel were evaluated.

## 2. Results and Discussion

### 2.1. Gel Formulations Based on Visual Consistency

The study aimed to develop a gel with the same appearance as a commercial gel. In this sense, several formulations were developed that fit the visual parameter of the consistency of the product. The standardized alcohol content (70% *w*/*v*) and the silver nanoparticles and saponins were varied to obtain the consistency closest to the control gel. Table 1 shows the formulations developed for subsequent evaluation.

### 2.2. pH and Organoleptic Evaluation

The pH test results of the gels are shown in Table 2. The results showed that there was a significant difference in all samples. It is recommended that the pH of the gels be proximate to the pH of the skin, which is 4.5–6.5 [45,46]. The results showed that most of the gels are close to this range, similar to those reported by Setiawan et al. [47]; the pH values could be influenced by the effect of the materials used for the preparation, according to Villa and Russo [24]. The pH of the gel with a high concentration of saponins and silver nanoparticles was higher in contrast with the other samples. Pérez Zamora et al. [48] reported that the pH values in gels with plant extracts and carbopol are close to neutrality (pH 6.4–6.9). The pH values of the gels developed in this study are in this range. Also, all values are higher than the control gel (commercial gel), probably attributable to the composition of the gel. The organoleptic evaluation was developed to establish if the use of artisan alcohol (*puntas smell*) presents a notable characteristic and to evaluate if the addition of silver nanoparticles and saponins affects the appearance of the gels. The visual appearance showed no differences in the appreciation of gel; it showed a clear gel with some bubbles and a fluid visual viscosity, similar to the control gel. On the other hand, the smell has a shallow level of persistence of the *puntas*, which is normal for this alcohol. Likewise, when the lumpiness was evaluated, there was no presence associated with the different formulations; lump presence was assessed as (++): high, (+): moderate, and (-) none.

### 2.3. Rheological Behavior

#### 2.3.1. Rotational Test

The most important properties of gels used as sanitizers are microbiological and rheological [42,49]. The viscosity and consistency depend on the network formed by the ingredients in the gel (use and concentration of different cross-linkers), which is reflected in the functionality and sensation it produces on the skin during use [50,51]. Efficiency, performance, and customer perception are linked to gel viscosity values. In the sensory aspect, the gel will be distributed on the skin for 20 to 30 s and dry entirely afterward [52]. For this reason, hydrogels can be considered more desirable than liquid forms due to the effortless application, alcoholic evaporation rate, and increased lethality in microorganisms [53].

**Figure 1:**
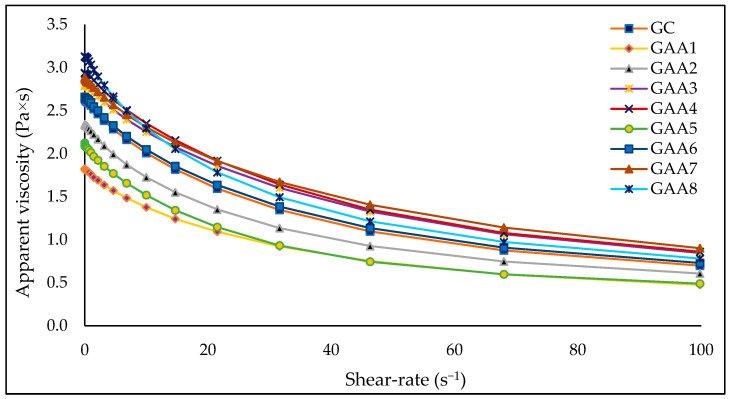
Relationship between apparent viscosity (Pa × s) and shear rate (s^−1^).

A clear decrease in the apparent viscosity was observed with increasing shear rate (Figure 1). The values of this property ranged between 1.806 and 3.121 Pa × s at 0.1 s^−1^, 1.377, and 2.352 Pa × s at 10 s^−1^, and 0.475 and 0.902 Pa × s at 100 s^−1^. Shukr and Ghada [54] reported an average of 0.304 Pa × s at the a shear rate and 1.43 Pa × s at a higher shear rate for similar gels made with 10% propylene glycol, 3% lemongrass oil, and different percentages of hydroxypropyl methylcellulose and sodium carboxymethyl cellulose. Meanwhile, samples GAA1 and GAA5 presented the lowest values in all the shear rate ranges. A more significant amount of ethyl alcohol (93.2 and 92.6%, respectively) considerably decreases the viscosity of the gels. The GAA6 formulation (ethyl alcohol 91%, carbopol 0.5%; triethanolamine 0.3%; propanediol 4.5%; silver nanoparticles 0.6%, and quinoa saponins 3.1%) presents a viscous behavior similar to the commercial gel (GC) used as a reference (Figure 1). The amount of ethyl alcohol used in the different formulations influences the viscosity values. In contrast, the amount of silver nanoparticles and quinoa saponins does not significantly change this physical parameter.

A study evaluated the influence of natural triterpenoid saponin (from the soapbark tree *Quillaja Saponaria* Molina) in emulsion gels. These samples showed a gradual decrease in apparent viscosity with an increasing shear rate from 0.1 to 100 s^−1^. Also, a dramatic reduction in apparent viscosity was observed with increased saponin concentration [55]. However, in this research, the amount of saponin used does not show a clear trend. This difference may be due to the purity of the saponin used in both studies. A correct gel viscosity allows the appropriate dose and a complete skin cover [56]. A reasonably high viscosity is desired to avoid wasting the gel when removing it from the container. The optimal viscosity values for a good hand sanitizer gel are 47 to 150 Pa × s [57,58]. The viscosity values presented by the gels in this research are below what was previously reported; however, in the sensory perception, they have a consistency similar to a typical commercial gel.

The rheological measurements can provide information related to the internal structure of gel components (as homogeneity/heterogeneity) [24]; in this case, the rheological behavior was measured by a rheometer using coaxial cylinders. The values obtained were adjusted to three equations describing viscous products’ rheological behavior. Determination coefficient values between 0.936 and 0.994 were obtained (Table 3). The highest values were obtained using power law and Herschel–Bulkley equations. The Herschel–Bulkley equation obtained extremely low yield stress (σ_0_) values. The results indicate that the gel is not a plastic fluid; therefore, it does not require a high initial force to start flowing. The flow behavior index (n) values reflect a deviation from Newtonian behavior. Values less than one show the pseudoplastic behavior of the gels.

**Figure 2:**
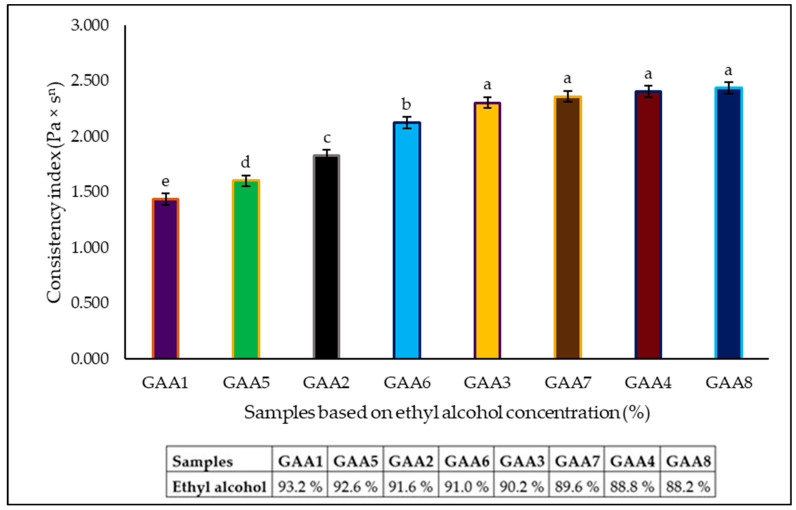
Consistency index (k) in Pa × s^n^ versus samples based on ethyl alcohol concentration (%). Different letters (a–e) in the same row indicate significant differences among samples (*p* ≤ 0.05).

Since the data fit better with the power law (Equation (1)), a statistical analysis was carried out to analyze the influence of the formulations on the rheological parameter k (consistency index). Higher values (*p* < 0.05) of k were observed in samples with lower ethyl alcohol concentration (Figure 2). An alcohol concentration above 90% significantly increased the pseudoplastic behavior of gels. No significant differences were evident between the samples GAA8, GAA4, GAA7, and GAA3; all these samples had alcohol concentrations under 90%.

#### 2.3.2. Oscillatory Test

**Figure 3:**
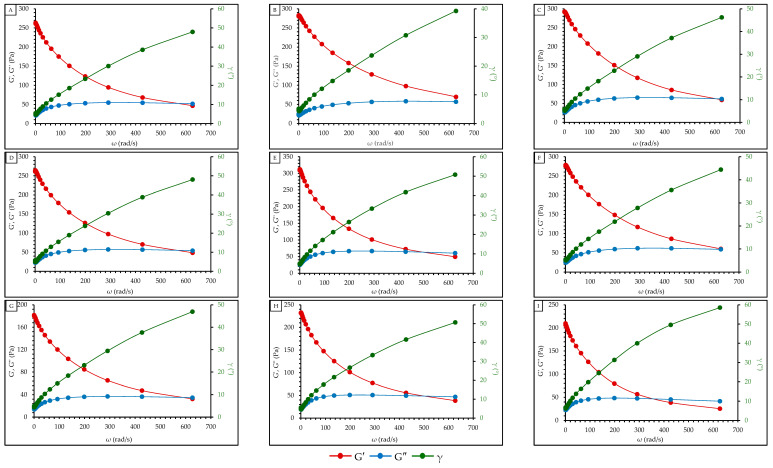
Storage modulus (G′), loss modulus (G″) in Pa × s. and phase angle (γ) in ° versus angular velocity (ω) in rad/s for the gels. (**A**) GC; (**B**) GAA1; (**C**) GAA2; (**D**) GAA3; (**E**) GAA4; (**F**) GAA5; (**G**) GAA6; (**H**) GAA7; (**I**) GAA8.

Oscillatory dynamic tests were performed to analyze the viscoelastic behavior of gels. The values of the elastic or storage modulus (G′), viscous or loss modulus (G″), and the loss tangent tan (δ) for each value of oscillating frequency (ω) are reported in Figure 3 for all the samples.

The value of the storage modulus (G′), loss modulus (G″) in Pa × s, and phase angle (γ) in grades (°) versus angular velocity (ω) in rad/s is graphed for all the samples (Figure 3). Note a decrease in G′ and an increase in G″ as a function of the rise in ω, indicating that the gel structure presents an increasingly viscous behavior with the execution of the experiment. Furthermore, the cut-off point between the module curves G′ (red line) and G″ (blue line) is observed. This point is known as crossover and indicates the point at which the gel has a phase angle equal to 45° [59]. At this point, the complete transition of the gel structure towards a downright viscous fluid occurs, completely losing its elastic component [60]. From a practical point of view, the consumer will perceive a more viscous sensation upon first contact with the gel, which will be lost in the distribution process on the skin. This loss of elasticity will enable better distribution and, therefore, better evaporation and adsorption. Most of the samples show the presence of crossover; however, in the sample GAA1, there is no evidence of crossover in the range of angular velocity (ω) worked.

**Figure 4:**
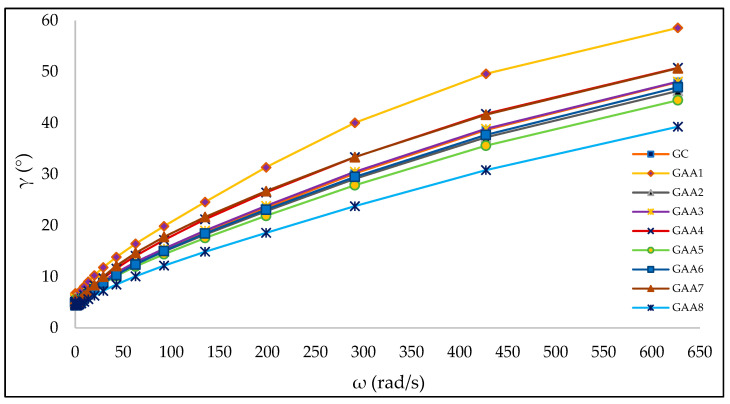
Phase angle (γ) in grades (°) versus angular velocity (ω) in rad/s for the gels.

The value of the phase angle (γ) in grades (°) is graphed as a function of the angular velocity (ω) in rad/s for all the formulations. Values of γ close to 0° represent more elastic than viscous behavior (typical behavior of solid materials). In comparison, values close to 90° represent more viscous than elastic behavior (typical behavior of liquid materials). The increasing trend observed in Figure 4 shows that the rheometer’s stress on the gels causes an internal deconfiguration, reflected in an increasingly viscous behavior. Also, results observed in Figure 4 shows higher phase angle (γ) values in sample GAA1 and lower values in sample GAA8. Once again, the influence of the alcohol percentage on the viscoelastic behavior of the gels is observed. Samples GAA2, GAA3, and GAA6 show a behavior similar to the commercial gel (GC) used as a reference. On the other hand, the storage modulus (G′) and the loss modulus (G″) did not show clear trends when plotted against the angular velocity (ω) in all the formulations.

### 2.4. Texture Profile Analysis

The gels were characterized concerning their hardness and elasticity (Table 4). Both properties showed significant differences (*p* < 0.05); the hardest gel was the control gel, and the gel produced with 40 mL of saponins and silver nanoparticles (GAA8) was the softest. A clear trend is not observed in all samples regarding the incorporation of saponins and silver particles; however, in the group of gels developed with artisan alcohol, it is observed that when the silver nanoparticles are included, and the concentration of saponins increases, the gel tends to be soft, similar to results reported by Martyasari et al. [61] in gels with Tekelan leaves extract. Although the results show a difference, it can be noted that the difference in hardness ranges around ~15% between the gels. This behavior could be attributed to the presence of electrolytes or an extreme pH, which could affect the texture of the gel. According to what was mentioned by Pérez Zamora, Michaluk, Torres, Mouriño, Chiappetta, and Nuñez [48], no specific behavior is observed regarding adding saponins as extracts. Concerning elasticity, defined as the ability of the deformed gel to recover its initial shape or length after the force has impacted it, it is observed that the control gel is less elastic in concordance with its high hardness. No specific trend is observed in all gels; however, in gels developed with artisanal alcohol, the least elastic samples correspond to the high concentration of saponins without silver nanoparticles added. The variation in terms of elasticity between the gels corresponds to ~20% of the gels produced in this study. This variation is significant and could be attributed to the nature of the artisan alcohol since the components present in the artisan alcohol, such as fermentation products, acids, or other components, reduce the elasticity of the gel [62].

A comparison between Figure 3 and Table 4 allows us to observe a relationship between the crossover values and elasticity. GAA1 is identified as the sample with the highest elasticity, and at the same time, it is the sample with no evidence of crossover in the range of angular velocity (ω) worked. From this, it can be inferred that the GAA1 gel has a higher water retention and lower retrogradation degree [63]. Contrary to this, the GAA8 sample shows the lowest elasticity, which shows the crossover at a lower value of ω (360 rad/s). This gel could show a lower structure ability to adapt to large strains, which results in more structural deformation and the appearance of viscous-like behavior [64].

### 2.5. Fourier-Transform Infrared Spectra of Gels

**Figure 5:**
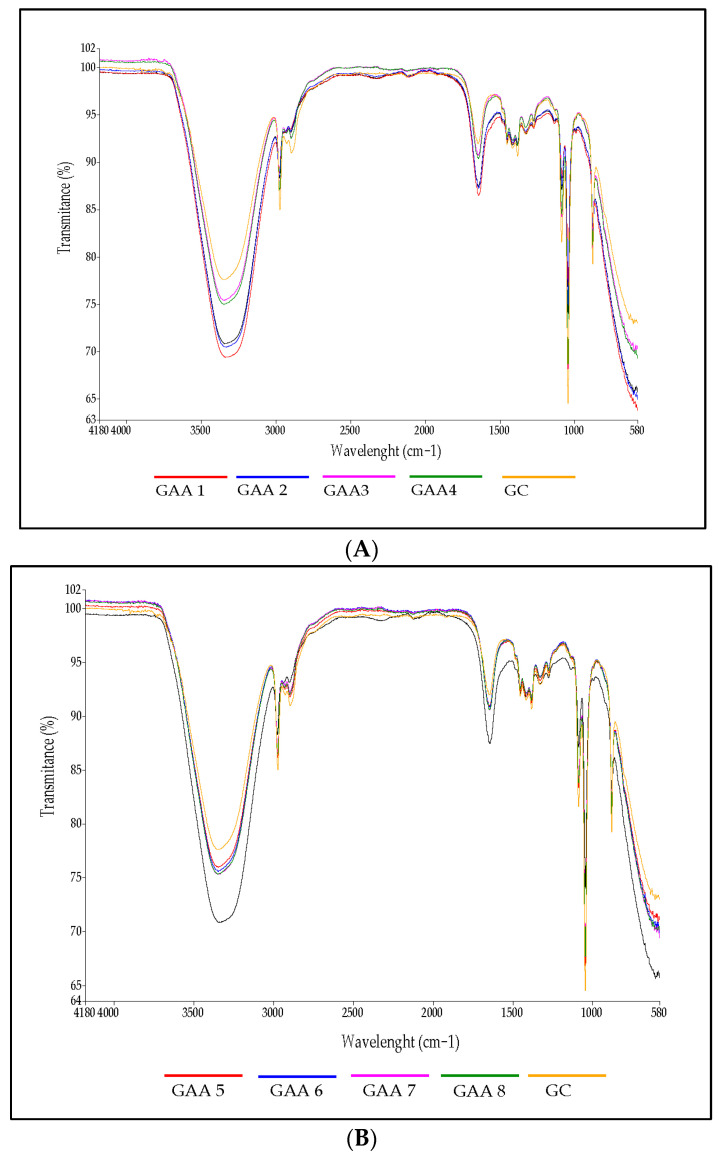
Fourier-transform infrared (FTIR) spectroscopy of the gels. (**A**) Gels developed with only saponins. (**B**) Gel developed with saponins and silver nanoparticles.

The IR spectrum of the gels is shown in Figure 5. The first dominant band from 3100 to 3500 cm^−1^ can be assigned to the extension of free and molecularly bound hydroxyl groups. Bands at 3000 and 2800 cm^−1^ were also observed, indicating extensions of the CH_2_ groups and the presence of lipids [65,66]. The band at 1650 cm^−1^ could correspond to the stretching of the C=O bond (C=O stretching) from the Amide I and to –OH, alkane groups (–CH, –CH_2_, –CH_3_) [67]. A band at 1417 cm^−1^ associated with the symmetric extension of the carboxyl group (–COO) was also observed [68]. The peak observed from 1000 to 1100 cm^−1^ corresponded to the ethereal crosslinking, representing a stretching vibration in the C–O–C group [69]. The peak of 878.23 cm^−1^ could be associated with C=CH compounds. The spectra do not indicate evidence of a difference in the presence of silver nanoparticles and saponins; the spectra show the strong signal attributed to a narrow peak corresponding to alcohol as a functional group that is located between 3230 and 3550 cm^−1^; the reduced concentration of saponins and silver nanoparticles probably do not generate a significant change that would allow any significant effect to be observed. These observations are similar to those reported in the literature [70,71].

### 2.6. Morphology by Scanning Electron Microscope (SEM)

**Figure 6:**
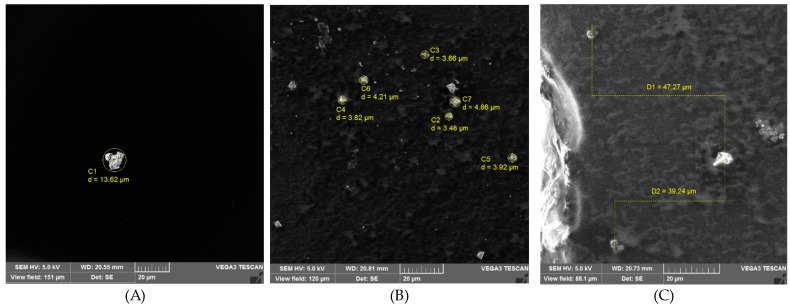
Micrographs obtained by SEM. (**A**) Saponins; (**B**) silver nanoparticles; (**C**) gel AA5.

Images of saponins, silver nanoparticles, and the gel AA5 are shown in Figure 6. Based on the observation that saponins have an irregular shape, the SEM photograph shows that the saponin’s size lies between 3.73 and 18.90 µm, while the silver nanoparticles’ size lies between 3.48 and 4.86 µm. The topography shows that saponins are irregular and have a rough external surface [72]. The saponins can create complexes that exhibit diverse shapes, including spherical, oblate, rod-shaped, lamellar, and worm-like structures [73]. In the case of silver nanoparticles, an external appearance with a smooth surface was observed, and a triangular and also quadrilateral shape were observed; the results of this study were similar to those reported by Thiruvengadam and Bansod [74], who reported a spherical shape in nature with some triangular morphology in silver nanoparticles synthesized using the chemical method. Finally, in the gel, it is possible to observe different particles associated with the saponins and silver nanoparticles, which are diffused in the gel matrix.

### 2.7. Antimicrobial Activity

**Figure 7:**
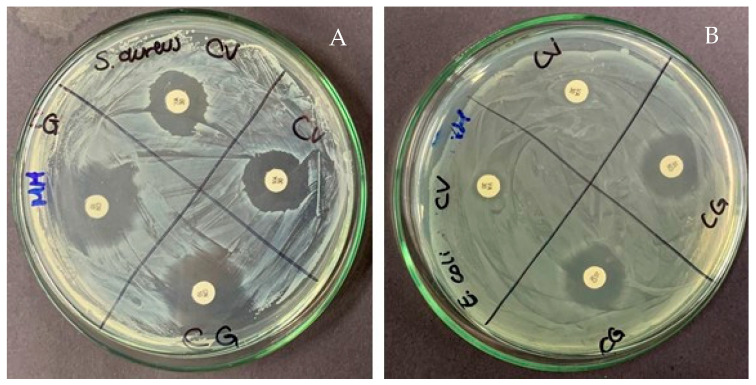
Inhibition halos of vancomycin (CV) and gentamicin (CG) as antimicrobials against Gram-negative (*E. coli*) and Gram-positive (*S. aureus*) bacteria. (**A**) *S. aureus* antimicrobial activity; (**B**) *E. Coli* antimicrobial activity.

The testing of the antimicrobial activity of formulations was developed according to the disk diffusion method [75]. The broad-spectrum antibacterial activity of gels was evaluated against Gram-positive bacteria *S. aureus* and Gram-negative bacteria *E. coli*. The zones of inhibition (mm) around the disk containing gels with silver nanoparticles and saponins are shown in Table 5. The antibacterial control media vancomycin (CV) and gentamicin (CG), which act as antimicrobials against Gram-negative bacteria (*E. coli*) and Gram-positive bacteria (*S. aureus*), respectively, showed their antibacterial effect by generating more giant halos [76,77] (Figure 7).

The antibacterial effect of the gels against *S. aureus* showed that gel GAA05 presented the greatest halo of inhibition compared to the other samples and the commercial gel; the remaining samples showed lower antibacterial effects. The results of the tested bacteria, *E. coli*, showed that the gel GAA05 has a higher inhibition zone, similar to those found with *S. aureus*. The low antimicrobial activity detected in most gels is presumed to correspond to a lower percentage of alcohol used in the formulation; as mentioned in previous lines, the objective of the work sought to establish formulations of visual viscosity similar to the control. The antimicrobial activity also increases when silver nanoparticles are included, in contrast with samples with only saponins [78,79].

On the other hand, the method used to extract saponins could be better; in this sense, it is essential to note that the content of saponins is in low concentration, attributable to the extraction method from quinoa [80]. The antibacterial activity found in the GAA05 sample could be attributed to the large surface area of silver nanoparticles, which could provide a larger contact surface with microorganisms [81,82]. Secondly, the antibacterial properties of silver nanoparticles as antibacterial agents [44,83] could increase the antibacterial effect of the alcohol. Third, a synergistic effect occurs between the silver particles and the added natural compounds [78].

## 3. Materials and Methods

Silver nanoparticles were purchased from La Casa del Químico, Quito, Ecuador (TNS Nanotechnology, Florianópolis, Brazil). Carbopol 940 was purchased from Alitecno (P) Ltd., Quito, Ecuador (Lubrizol, Wickliffe, OH, USA). The Mueller–Hinton agar, 0.5 McFarland standards, was purchased from Environobolab Ltd., Quito, Ecuador. Analytical grade solvents and chemicals were used in their purest form and were not further purified.

### 3.1. Gel preparation Process

The preparation of the antibacterial gel was based on a study proposed by Tripathy [18]. The hydrophilic carbopol was sieved and subsequently poured into distilled water and stirred constantly for approximately 10 min to form a homogeneous mixture, avoiding the formation of lumps.

Next, 30% of the alcohol (70%) was poured in with constant stirring for 20 min, the remaining 70% of the alcohol was added, and the same stirring process was repeated; 1,2-propanediol was added until gelation. Finally, the silver nanoparticles and saponins were added. The size of silver nanoparticles was 15 nm, and they were stabilized with organic molecules. To stabilize the pH range of 6 to 7, triethanolamine (TEA) was added.

### 3.2. pH and Organoleptical Determinations

A visual organoleptic test was conducted to observe the shape and color, and the smell of the gel preparations was developed. The pH of the gels was determined following the methodology described by Alqarni et al. [84]. The tests were carried out in triplicate.

### 3.3. Rheological Behavior

#### 3.3.1. Rotational Test

The flow curves of gels were determined using an Anton Paar MCR 302 rheometer (Anton Paar GmbH, Graz, Austria) with a coaxial cylinder geometry. The system’s temperature was set and maintained at 20 °C through a circulator water bath (DC30, Haake, Karlsruhe, Germany). The flow curves of the gels were determined using a shear rate from 0.01 to 100 s^−1^—first ascending, then descending, and finally ascending again—to eliminate possible thixotropy in the sample. The rheological properties were fitted to the Power law, Herschel–Bulkley, and Casson models (Table 6). The average and standard deviation of six repetitions are presented.

#### 3.3.2. Oscillatory Test

To analyze the viscoelastic behavior of gels, oscillatory dynamic tests were performed in an Anton Paar MCR 302 rheometer (Anton Paar GmbH, Austria), with a system of parallel plates (25 mm diameter) and Peltier temperature control (20 °C), according to the methodology reported by Li, Zhao and Chen [88]. For each value of oscillating frequency (ω), values for the elastic or storage modulus (G′), viscous or loss modulus (G″), and the loss tangent tan (δ) were obtained.

### 3.4. Texture Profile Analysis

Texture profile analysis (TPA) was performed following the method proposed by Pérez Zamora, Michaluk, Torres, Mouriño, Chiappetta, and Nuñez [48]; the measurements were performed using a Brookfield CT3 texturometer with the TA11/1000 probe. The hardness and elasticity of the gels were evaluated. The test parameters were the following: load cell 4500 g, activation load 1 g, test speed 1 mm/s, return speed 1 mm/s, cycles: 1. The cylinder (sample container) was 30 mm high and 38 mm in diameter.

### 3.5. Fourier-Transform Infrared Spectra of Gels

Infrared spectroscopy is one of the most important analytical methods for detecting certain functional groups of polymers and drugs; the evaluation method was proposed by Beć et al. [89]. The infrared spectra of the gels were evaluated using an infrared spectrophotometer (Perkin–Elmer, model Spectrum Two, Columbus, OH, USA) covering wave numbers from 4000 to 500 cm^−1^. The measurements were made in triplicate.

### 3.6. Scanning Electron Microscope (SEM)

The morphology of the gels was performed in a scanning electron microscope (SEM) (Vega3, Tescan, Warrendale, PA, USA) with an accelerated voltage of 100 Kv. The samples were air-dried and then sputter-coated with gold. Finally, each sample was observed at 5 Kv in the microscope, with a field of view of 253 μm and a magnification of 546× *g*.

### 3.7. Antimicrobial Activity

#### 3.7.1. Microorganisms

The Gram-positive bacteria (*Staphylococcus aureus* (*S. aureus*) ATCC 6538) and Gram-negative bacteria (*Escherichia coli* (*E. coli*) ATCC 8739) used in this study were obtained from the American Type Culture Collection (ATCC). The bacterial strains were grown at 37 °C and maintained on nutrient agar.

#### 3.7.2. Well-Diffusion Method

The antimicrobial activity of gels was tested in vitro against Gram-positive and Gram-negative bacteria using the method proposed by Bauer et al. [90]. The medium used was Mueller–Hinton agar. The bacterial inoculum was prepared in 5 mL of phosphate-buffered saline (0.5 McFarland standards), where 100 µL of bacterial suspension was inoculated on fresh Mueller–Hinton agar. Wells were then bored on the Muller–Hinton agar plates using a sterilized borer. Each well was filled with 30 µL of prepared gels. Vancomycin (CV) and gentamicin (CG), which act as antimicrobials against Gram-negative (*E. coli*) and Gram-positive (*S. aureus*) bacteria, were considered the control agents. The inoculated plates with pathogenic bacteria were incubated at 37 °C for 18 to 24 h. The test was repeated twice for each sample.

### 3.8. Statistical Analysis

The results were analyzed by one-way ANOVA, and means were tested using Tukey’s multiple comparisons to investigate the statistical significance. All statistical analyses with a significance level α = 0.05 were performed using Prism-GraphPad v5.03 (GraphPad Software, Inc., San Diego, CA, USA).

## 4. Conclusions

The results obtained in this study allowed us to establish that the use of artisanal alcohol for the development of sanitary gels is a viable alternative. Likewise, including saponins from quinoa and silver nanoparticles increases the gel’s antimicrobial power. The antibacterial efficacy observed in sample GAA05 may be due to several factors, such as a high alcohol concentration in the formulation and the synergistic effect between silver particles and saponins. The evaluated properties allowed us to establish that each gel has a different behavior, mainly associated with the formulation developed, which sought to simulate the viscosity of the commercial gel.

## Figures and Tables

**Table 1:** Formulations of the gel developed with artisan alcohol.

Components	Samples							
(%)	GAA1	GAA2	GAA3	GAA4	GAA5	GAA6	GAA7	GAA8
Ethyl alcohol	93.2	91.6	90.2	88.8	92.6	91.0	89.6	88.2
Carbopol	0.5	0.5	0.5	0.5	0.5	0.5	0.5	0.5
Triethanolamine	0.3	0.3	0.3	0.3	0.3	0.3	0.3	0.3
Propanediol	4.5	4.5	4.5	4.5	4.5	4.5	4.5	4.5
Silver nanoparticles	-	-	-	-	0.6	0.6	0.6	0.6
Quinoa saponins	1.5	3.1	4.5	5.9	1.5	3.1	4.5	5.9

**Table 2:** pH and organoleptically results of the gel.

Samples	pH	Visual Appearance	Visual Viscosity	Smell Characteristic	Lumps
GC	6.30 ± 0.01 ^e^	Clear + Bubbles	Semi fluid	Alcohol	-
GAA1	6.58 ± 0.02 ^c^	Clear + Bubbles	Semi fluid	Alcohol + *Puntas*	-
GAA2	6.40 ± 0.02 ^d^	Clear + Bubbles	Semi fluid	Alcohol + *Puntas*	-
GAA3	6.58 ± 0.01 ^c^	Clear + Bubbles	Semi fluid	Alcohol + *Puntas*	-
GAA4	6.69 ± 0.01 ^b^	Clear + Bubbles	Semi fluid	Alcohol + *Puntas*	-
GAA5	6.68 ± 0.02 ^b^	Clear + Bubbles	Semi fluid	Alcohol + *Puntas*	-
GAA6	6.38 ± 0.01 ^d^	Clear + Bubbles	Semi fluid	Alcohol + *Puntas*	-
GAA7	6.58 ± 0.01 ^c^	Clear + Bubbles	Semi fluid	Alcohol + *Puntas*	-
GAA8	6.75 ± 0.01 ^a^	Clear + Bubbles	Semi fluid	Alcohol + *Puntas*	-

The results are the mean ± standard deviation. One-way ANOVA: Different letters (a–e) in the same row indicate significant differences among samples (*p* ≤ 0.05).

**Table 3:** Parameters obtained in the rheological mathematical modeling.

Treatments		Models		
	Model Constants and r^2^ Adjustment	Power Law $\sigma =k{\left(\dot{\gamma }\right)}^{n}$	Herschel–Bulkley $\sigma =\sigma_{0}+k{\left(\dot{\gamma }\right)}^{n}$	Casson $\sigma^{0.5}={\sigma_{0}}^{0.5}+k{\left(\dot{\gamma }\right)}^{0.5}$
GC	Model constants	k: 2.093 n: 0.894	σ_0_: 4.472 × 10^−4^ k: 2.088 n: 0.870	σ_0_: 0.769 k: 0.933
	Adj. r^2^	0.992	0.992	0.945
GAA1	Model constants	k: 1.437 n: 0.893	σ_0_: 4.789 × 10^−5^ k: 1.437 n: 0.868	σ_0_: 0.702 k: 0.771
	Adj. r^2^	0.992	0.992	0.945
GAA2	Model constants	k: 1.830 n: 0.889	σ_0_: 3.975 × 10^−4^ k: 1.826 n: 0.863	σ_0_: 0.748 k: 0.862
	Adj. r^2^	0.991	0.991	0.946
GAA3	Model constants	k: 2.303 n: 0.909	σ_0_: 1.387 × 10^−4^ k: 2.302 n: 0.887	σ_0_: 0.755 k: 1.029
	Adj. r^2^	0.993	0.993	0.953
GAA4	Model constants	k: 2.404 n: 0.906	σ_0_: 3.734 × 10^−4^ k: 2.400 n: 0.883	σ_0_: 0.774 k: 1.038
	Adj. r^2^	0.993	0.993	0.951
GAA5	Model constants	k: 1.602 n: 0.877	σ_0_: 1.839 × 10^−4^ k: 1.600 n: 0.850	σ_0_: 0.748 k: 0.773
	Adj. r^2^	0.990	0.990	0.936
GAA6	Model constants	k: 2.124 n: 0.898	σ_0_: 4.992 × 10^−4^ k: 2.119 n: 0.874	σ_0_: 0.764 k: 0.952
	Adj. r^2^	0.992	0.992	0.948
GAA7	Model constants	k: 2.360 n: 0.913	σ_0_: 4.803 × 10^−4^ k: 2.355 n: 0.894	σ_0_: 0.745 k: 1.061
	Adj. r^2^	0.994	0.994	0.958
GAA8	Model constants	k: 2.437 n: 0.886	σ_0_: 2.194 × 10^−4^ k: 2.435 n: 0.859	σ_0_: 0.812 k: 0.982
	Adj. r^2^	0.991	0.991	0.943

σ_0_: yield stress (Pa); k: consistency index (Pa × s^n^); n: flow behavior index (dimensionless).

**Table 4:** Textural parameters of the gels.

Sample	Hardness (g)	Elasticity (mm)
GC	113.02 ± 0.03 ^a^	7.47 ± 0.05 ^f^
GAA1	101.00 ± 0.04 ^b^	8.07 ± 0.04 ^e^
GAA2	89.00 ± 0.01 ^e^	9.74 ± 0.05 ^a^
GAA3	96.00 ± 0.04 ^c^	7.86 ± 0.03 ^g^
GAA4	89.00 ± 0.03 ^e^	9.22 ± 0.02 ^b^
GAA5	93.00 ± 0.04 ^d^	9.32 ± 0.03 ^b^
GAA6	88.00 ± 0.03 ^f^	9.81 ± 0.03 ^a^
GAA7	87.00 ± 0.02 ^g^	8.29 ± 0.04 ^d^
GAA8	86.00 ± 0.02 ^h^	8.96 ± 0.02 ^c^

The results are the mean ± standard deviation. One-way ANOVA: Different letters (a–h) in the same row indicate significant differences among samples (*p* ≤ 0.05).

**Table 5:** Inhibition halos of studied formulations.

Samples	*S. aureus* (mm)	*E. coli* (mm)
GC	9.75 ± 0.02 ^b^	11.50 ± 0.02 ^b^
GAA1	7.50 ± 0.03 ^g^	8.75 ± 0.03 ^g^
GAA2	9.00 ± 0.01 ^d^	9.00 ± 0.01 ^f^
GAA3	9.75 ± 0.03 ^b^	8.00 ± 0.03 ^h^
GAA4	8.75 ± 0.04 ^e^	10.75 ± 0.04 ^c^
GAA5	11.50 ± 0.02 ^a^	12.12 ± 0.02 ^a^
GAA6	8.75 ± 0.04 ^e^	9.00 ± 0.04 ^f^
GAA7	8.00 ± 0.02 ^f^	9.25 ± 0.02 ^e^
GAA8	9.62 ± 0.03 ^c^	10.62 ± 0.03 ^d^

The results are the mean ± standard deviation. One-way ANOVA: Different letters (a–h) in the same row indicate significant differences among samples (*p* ≤ 0.05).

**Table 6:** Equations used for modeling the rheological behavior.

Model	Equation	Equation Number	References
Power law	$\sigma =k{\left(\dot{\gamma }\right)}^{n}$	(1)	[85]
Herschel–Bulkley	$\sigma =\sigma_{0}+k{\left(\dot{\gamma }\right)}^{n}$	(2)	[86]
Casson	$\sigma^{0.5}={\sigma_{0}}^{0.5}+k{\left(\dot{\gamma }\right)}^{0.5}$	(3)	[87]

where *σ* is the shear stress (Pa), σ_0_ is the yield stress, $\dot{\gamma }$ is the shear rate (s^−1^), *k* is the consistency index (Pa × s^n^), and *n* is the flow behavior index (dimensionless).

## Data Availability

Data are contained within the article.

## References

[1] Ariyanthini K.S., Angelina E., Permana K.N.B., Thelmalina F.J., Prasetia I. (2021). Antibacterial activity testing of hand sanitizer gel extract of coriander (*Coriandrum sativum* L.) Seeds against Staphylococcus aureus. J. Pharm. Sci. Appl..

[2] Ma Y., Yi J., Ma J., Yu H., Luo L., Wu W., Jin L., Yang Q., Lou T., Sun D. (2023). Hand sanitizer gels: Classification, challenges, and the future of multipurpose hand hygiene products. Toxics.

[3] Imani S.M., Ladouceur L., Marshall T., Maclachlan R., Soleymani L., Didar T.F. (2020). Antimicrobial nanomaterials and coatings: Current mechanisms and future perspectives to control the spread of viruses including SARS-CoV-2. ACS Nano.

[4] Mallakpour S., Azadi E., Hussain C.M. (2021). Protection, disinfection, and immunization for healthcare during the COVID-19 pandemic: Role of natural and synthetic macromolecules. Sci. Total Environ..

[5] Ontong J.C., Singh S., Nwabor O.F., Chusri S., Voravuthikunchai S.P. (2020). Potential of antimicrobial topical gel with synthesized biogenic silver nanoparticle using *Rhodomyrtus tomentosa* leaf extract and silk sericin. Biotechnol. Lett.

[6] Rai N., Shukla T.P., Loksh K.R., Karole S. (2020). Synthesized silver nanoparticle loaded gel of Curcuma caesia for effective treatment of acne. J. Drug. Del. Ther. Clin. Risk Manag..

[7] Islam M.A., Jacob M.V., Antunes E. (2021). A critical review on silver nanoparticles: From synthesis and applications to its mitigation through low-cost adsorption by biochar. J. Environ. Manag..

[8] Yin I.X., Zhang J., Zhao I.S., Mei M.L., Li Q., Chu C.H. (2020). The Antibacterial Mechanism of Silver Nanoparticles and Its Application in Dentistry. Int. J. Nanomed..

[9] Gashaw G., Fassil A., Redi F. (2020). Evaluation of the antibacterial activity of *Pleurotus* spp. cultivated on different agricultural wastes in Chiro, Ethiopia. Int. J. Microbiol..

[10] Dwivedi M.K., Sonter S., Mishra S., Patel D.K., Singh P.K. (2020). Antioxidant, antibacterial activity, and phytochemical characterization of *Carica papaya* flowers. Beni-Suef Univ. J. Basic Appl. Sci..

[11] Chojnacka K., Witek-Krowiak A., Skrzypczak D., Mikula K., Młynarz P. (2020). Phytochemicals containing biologically active polyphenols as an effective agent against COVID-19-inducing coronavirus. J. Funct. Foods.

[12] Huynh N.K., Nguyen D.H.M., Nguyen H.V.H. (2022). Effects of processing on oxalate contents in plant foods: A review. J. Food Compost. Anal..

[13] Takahashi C., Vílchez H., Poemape J., Alvia A., Olortegui A. (2023). Diversity of bioactive compounds from *Tropaeolum tuberosum* (mashua). Rev. Colomb. Cienc. Quim. Farm..

[14] Acurio L.P., Salazar D.M., Valencia A.F., Robalino D.R., Barona A.C., Alvarez F.C., Rodriguez C.A. (2018). Antimicrobial potential of *Chlorella* algae isolated from stacked waters of the Andean Region of Ecuador. IOP Conf. Ser. Earth Environ. Sci..

[15] Little S.M., Senhorinho G.N.A., Saleh M., Basiliko N., Scott J.A. (2021). Antibacterial compounds in green microalgae from extreme environments: A review. Algae.

[16] Syukri D.M., Nwabor O.F., Singh S., Ontong J.C., Wunnoo S., Paosen S., Munah S., Voravuthikunchai S.P. (2020). Antibacterial-coated silk surgical sutures by ex situ deposition of silver nanoparticles synthesized with *Eucalyptus camaldulensis* eradicates infections. J. Microbiol. Methods.

[17] Tummino M.L., Laurenti E., Bracco P., Cecone C., Parola V.L., Vineis C., Testa M.L. (2023). Antibacterial properties of functionalized cellulose extracted from deproteinized soybean hulls. Cellulose.

[18] Tripathy D., Gadtya A.S., Moharana S. (2023). Supramolecular Gel, Its classification, preparation, properties, and applications: A review. Polym.-Plast. Technol. Mater..

[19] Talat M., Zaman M., Khan R., Jamshaid M., Akhtar M., Mirza A.Z. (2021). Emulgel: An effective drug delivery system. Drug Dev. Ind. Pharm..

[20] Mohd Shahrizan M.S., Abd Aziz Z.H., Katas H. (2022). Fluid gels: A systematic review towards their application in pharmaceutical dosage forms and drug delivery systems. J. Drug Deliv. Sci. Technol..

[21] Martín-Illana A., Notario-Pérez F., Cazorla-Luna R., Ruiz-Caro R., Bonferoni M.C., Tamayo A., Veiga M.D. (2022). Bigels as drug delivery systems: From their components to their applications. Drug Discov. Today.

[22] Ho T.-C., Chang C.-C., Chan H.-P., Chung T.-W., Shu C.-W., Chuang K.-P., Duh T.-H., Yang M.-H., Tyan Y.-C. (2022). Hydrogels: Properties and Applications in Biomedicine. Molecules.

[23] Suchomel M., Steinmann J., Kampf G. (2020). Efficacies of the original and modified World Health Organization-recommended hand-rub formulations. J. Hosp. Infect..

[24] Villa C., Russo E. (2021). Hydrogels in Hand Sanitizers. Materials.

[25] Boyce J., Chartier Y., Chraiti M., Cookson B., Damani N., Dharan S. WHO. Guidelines on Hand Hygiene in Health Care. First Global Patient Safety Challenge Clean Care Is Safer Care. https://pubmed.ncbi.nlm.nih.gov/23805438/.

[26] Aswathy S., Narendrakumar U., Manjubala I. (2020). Commercial hydrogels for biomedical applications. Heliyon.

[27] Berardi A., Perinelli D.R., Merchant H.A., Bisharat L., Basheti I.A., Bonacucina G., Cespi M., Palmieri G.F. (2020). Hand sanitisers amid COVID-19: A critical review of alcohol-based products on the market and formulation approaches to respond to increasing demand. Int. J. Pharm..

[28] Lin Q., Lim J.Y., Xue K., Yew P.Y.M., Owh C., Chee P.L., Loh X.J. (2020). Sanitizing agents for virus inactivation and disinfection. View.

[29] Hans M., Lugani Y., Chandel A.K., Rai R., Kumar S. (2023). Production of first-and second-generation ethanol for use in alcohol-based hand sanitizers and disinfectants in India. Biomass Convers. Biorefin.

[30] Monsalve M.S. (2020). Bebidas ancestrales y tradicionales de Colombia (Región del Pacífico). Sosquua.

[31] Valdés-Solano D.M., Fonseca-Herreño L.C., Alba-Maldonado J.M. (2020). Diagnostic tools for measuring the manufacture of Colombian traditional emerging products. J. Phys. Conf. Ser..

[32] Viana E.J., de Carvalho Tavares I.M., Rodrigues L.M.A., das Graças Cardoso M., Júnior J.C.B., Gualberto S.A., de Oliveira C.P. (2020). Evaluation of toxic compounds and quality parameters on the aged Brazilian sugarcane spirit. Res. Soc. Dev. Chang..

[33] Singh S., Roy S., Prasad L., Chauhan A.S. (2023). Bio-Alcohol. Biofuel Extraction Techniques.

[34] Aguilar-Rivera N., Olvera-Vargas L.A. (2023). Impacts of public policies and stakeholders in the transition from the biofuel value chain to a circular bioeconomy: México as a case study. Advances in Lignocellulosic Biofuel Production Systems.

[35] Hendriks H. (2020). Alcohol and human health: What is the evidence?. Annu. Rev. Food Sci. Technol..

[36] Tse T.J., Wiens D.J., Reaney M.J. (2021). Production of bioethanol—A review of factors affecting ethanol yield. Fermentation.

[37] Edeh I. (2021). Bioethanol production: An overview. J. Bioeth. Tech..

[38] Liu C.-G., Li K., Wen Y., Geng B.-Y., Liu Q., Lin Y.-H. (2019). Bioethanol: New opportunities for an ancient product. Advanced Bioenergy.

[39] Erasmus V., Daha T.J., Brug H., Richardus J.H., Behrendt M.D., Vos M.C., van Beeck E.F. (2010). Systematic review of studies on compliance with hand hygiene guidelines in hospital care. Infect. Control Hosp. Epidemiol..

[40] Mahmood A., Eqan M., Pervez S., Alghamdi H.A., Tabinda A.B., Yasar A., Brindhadevi K., Pugazhendhi A. (2020). COVID-19 and frequent use of hand sanitizers; human health and environmental hazards by exposure pathways. Sci. Total Environ..

[41] Dal’Belo S.E., Rigo Gaspar L., Berardo Gonçalves Maia Campos P.M. (2006). Moisturizing effect of cosmetic formulations containing Aloe vera extract in different concentrations assessed by skin bioengineering techniques. Skin. Res. Technol..

[42] Booq R.Y., Alshehri A.A., Almughem F.A., Zaidan N.M., Aburayan W.S., Bakr A.A., Kabli S.H., Alshaya H.A., Alsuabeyl M.S., Alyamani E.J. (2021). Formulation and Evaluation of Alcohol-Free Hand Sanitizer Gels to Prevent the Spread of Infections during Pandemics. Int. J. Env. Res. Public Health.

[43] Fallica F., Leonardi C., Toscano V., Santonocito D., Leonardi P., Puglia C. (2021). Assessment of Alcohol-Based Hand Sanitizers for Long-Term Use, Formulated with Addition of Natural Ingredients in Comparison to WHO Formulation 1. Pharmaceutics.

[44] Dong S., Yang X., Zhao L., Zhang F., Hou Z., Xue P. (2020). Antibacterial activity and mechanism of action saponins from Chenopodium quinoa Willd. husks against foodborne pathogenic bacteria. Ind. Crops Prod..

[45] Das S., Wong A.B. (2020). Stabilization of ferulic acid in topical gel formulation via nanoencapsulation and pH optimization. Sci. Rep. Cetacean Res..

[46] Sitohang N.A., Putra E.D. (2023). Effect of various concentrations of *Barringtonia racemosa* (l). Spreng extract on Physical stability of Topical gel. Res. J. Pharm. Technol..

[47] Setiawan B., Fika R., Fadhila M., Trisna M., Putri L.A. (2023). Effect of Different Concentrations of Propylene Glycol and Glycerin on the Formulation of Guava Leaf (Psidium Guajava Linn.) Body Scrub with White Rice (Oryza sativa Linn.). Jurnal Eduhealth.

[48] Pérez Zamora C.M., Michaluk A.G., Torres C.A., Mouriño V.S.L., Chiappetta D.A., Nuñez M.B. (2023). Influence of herbal extracts in physicochemical properties and stability of antibacterial gels. J. Adv. Pharm. Educ. Res..

[49] Perinelli D.R., Berardi A., Bisharat L., Cambriani A., Ganzetti R., Bonacucina G., Cespi M., Palmieri G.F. (2021). Rheological properties of cellulosic thickeners in hydro-alcoholic media: The science behind the formulation of hand sanitizer gels. Int. J. Pharm..

[50] Berardi A., Perinelli D.R., Bisharat L., Sabbatini B., Bonacucina G., Tiboni M., Palmieri G.F., Cespi M. (2022). Factors affecting the rheological behaviour of carbomer dispersions in hydroalcoholic medium: Towards the optimization of hand sanitiser gel formulations. Int. J. Pharm..

[51] Mohite P.B., Adhav S.S. (2017). A hydrogels: Methods of preparation and applications. Int. J. Adv. Pharm.

[52] Sommatis S., Capillo M.C., Maccario C., Rauso R., D’Este E., Herrera M., Castiglioni M., Mocchi R., Zerbinati N. (2023). Antimicrobial efficacy assessment and rheological investigation of two different hand sanitizers compared with the standard reference WHO formulation 1. Gels.

[53] Silva A.F., Wood T.A., Hodgson D.J., Royer J.R., Thijssen J.H., Lips A., Poon W.C. (2022). Rheological design of thickened alcohol-based hand rubs. Rheol. Acta.

[54] Shukr M., Metwally G.F. (2013). Evaluation of topical gel bases formulated with various essential oils for antibacterial activity against methicillin-resistant Staphylococcus aureus. Trop. J. Pharm. Res..

[55] Yin W.-J., Chen X.-W., Ma C.-G., Wang J.-M. (2022). Fabrication and characterization of tunable high internal phase emulsion gels (HIPE-Gels) formed by natural triterpenoid saponin and plant soy protein. ACS Food Sci..

[56] Miastkowska M., Kulawik-Pióro A., Szczurek M. (2020). Nanoemulsion gel formulation optimization for burn wounds: Analysis of rheological and sensory properties. Processes.

[57] Surini S., Amirtha N.I., Lestari D.C. (2018). Formulation and effectiveness of a hand sanitizer gel produced using salam bark extract. Int. J. Appl. Pharm..

[58] Lieberman H., Rieger M., Banker G.S. (2020). Pharmaceutical Dosage Forms: Disperse Systems.

[59] Bonacucina G., Spina M., Misici-Falzi M., Cespi M., Pucciarelli S., Angeletti M., Palmieri G.F. (2007). Effect of hydroxypropyl β-cyclodextrin on the self-assembling and thermogelation properties of Poloxamer 407. Eur. J. Pharm. Sci..

[60] Winter H.H. (1987). Can the gel point of a cross-linking polymer be detected by the G′-G″ crossover?. Polym. Eng. Sci..

[61] Martyasari N.W.R., Andayani Y., Hajrin W. (2019). Optimisation of hand sanitiser gel formula of Tekelan leaves extract (*Chromolaena odorata*) using simplex lattice design method. Bali Med. J..

[62] Golin A.P., Choi D., Ghahary A. (2020). Hand sanitizers: A review of ingredients, mechanisms of action, modes of delivery, and efficacy against coronaviruses. Am. J. Infect. Control.

[63] Hurler J., Engesland A., Poorahmary Kermany B., Škalko-Basnet N. (2012). Improved texture analysis for hydrogel characterization: Gel cohesiveness, adhesiveness, and hardness. J. Appl. Polym. Sci..

[64] Anvari M., Joyner H.S. (2017). Effect of formulation on structure-function relationships of concentrated emulsions: Rheological, tribological, and microstructural characterization. Food Hydrocoll..

[65] Hamed R., Abu Alata W.a., Abu-Sini M., Abulebdah D.H., Hammad A.M., Aburayya R. (2023). Development and comparative evaluation of ciprofloxacin nanoemulsion-loaded bigels prepared using different ratios of oleogel to hydrogels. Gels.

[66] Ramírez Martínez C. (2022). Synthesis and Characterization of a Natural-Based Hydrogel for Biomedical Applications. https://repositorio.tec.mx/handle/11285/650868.

[67] Teoh W.K., Muslim N.Z.M., Ismail M.L., Chang K.H., Abdullah A.F.L. (2021). Quick determination and discrimination of commercial hand sanitisers using attenuated total reflectance-Fourier transform infrared spectroscopy and chemometrics. Anal. Methods.

[68] El-Sakhawy M., Tohamy H.-A.S., AbdelMohsen M.M., El-Missiry M. (2023). Biodegradable carboxymethyl cellulose based material for sustainable/active food packaging application. J. Thermoplast. Compos. Mater..

[69] Xie D., Jiang Y., Xu R., Zhang Z., Chen G. (2023). Preparation of ethanol-gels as hand sanitizers formed from chitosan and silica nanoparticles. J. Mol. Liq..

[70] Ahmadi P., Jahanban-Esfahlan A., Ahmadi A., Tabibiazar M., Mohammadifar M. (2022). Development of ethyl cellulose-based formulations: A perspective on the novel technical methods. Food Rev. Int..

[71] Hamed R., AbuRezeq A., Tarawneh O. (2018). Development of hydrogels, oleogels, and bigels as local drug delivery systems for periodontitis. Drug Dev. Ind. Pharm..

[72] García-Salcedo Á.J., Torres-Vargas O.L., Ariza-Calderón H. (2018). Physical-chemical characterization of quinoa (*Chenopodium quinoa* Willd.), amaranth (*Amaranthus caudatus* L.), and chia (*Salvia hispanica* L.) flours and seeds. Acta Agronon..

[73] Liao Y., Li Z., Zhou Q., Sheng M., Qu Q., Shi Y., Yang J., Lv L., Dai X., Shi X. (2021). Saponin surfactants used in drug delivery systems: A new application for natural medicine components. Int. J. Pharm..

[74] Thiruvengadam V., Bansod A. (2020). Characterization of silver nanoparticles synthesized using chemical method and its antibacterial property. Biointerface Res. Appl. Chem..

[75] Wanger A., Chávez V. (2021). Antibiotic susceptibility testing. Practical Handbook of Microbiology.

[76] Kachur K., Suntres Z. (2020). The antibacterial properties of phenolic isomers, carvacrol and thymol. Crit. Rev. Food Sci. Nutr..

[77] Strahilevitz J., Rubinstein E. (2002). Novel agents for resistant Gram-positive infections—A review. Int. J. Infect. Dis..

[78] Durán N., Durán M., de Jesus M.B., Seabra A.B., Fávaro W.J., Nakazato G. (2016). Silver nanoparticles: A new view on mechanistic aspects on antimicrobial activity. Nanomed. Nanotechnol. Biol. Med..

[79] Prasad S.R., Teli S.B., Ghosh J., Prasad N.R., Shaikh V.S., Nazeruddin G.M., Al-Sehemi A.G.A., Patel I., Shaikh Y.I. (2021). A Review on Bio-inspired Synthesis of Silver Nanoparticles: Their Antimicrobial Efficacy and Toxicity. Eng. Sci..

[80] Mora-Ocación M.S., Morillo-Coronado A.C., Manjarres-Hernández E.H. (2022). Extraction and Quantification of Saponins in Quinoa (*Chenopodium quinoa* Willd.) Genotypes from Colombia. Int. J. Food Sci..

[81] Anju T.R., Parvathy S., Valiya Veettil M., Rosemary J., Ansalna T.H., Shahzabanu M.M., Devika S. (2021). Green synthesis of silver nanoparticles from Aloe vera leaf extract and its antimicrobial activity. Mater. Today Proc..

[82] Chinnasamy G., Chandrasekharan S., Koh T.W., Bhatnagar S. (2021). Synthesis, Characterization, Antibacterial and Wound Healing Efficacy of Silver Nanoparticles from Azadirachta indica. Front. Microbiol..

[83] Amraei S., Ahmadi S. (2022). Recent studies on antimicrobial and anticancer activities of saponins: A mini-review. Nano Micro Biosyst..

[84] Alqarni M.H., Foudah A.I., Alam A., Salkini M.A., Muharram M.M., Labrou N.E., Kumar P. (2022). Development of gum-acacia-stabilized silver nanoparticles gel of rutin against Candida albicans. Gels.

[85] Song K.-W., Kim Y.-S., Chang G.-S. (2006). Rheology of concentrated xanthan gum solutions: Steady shear flow behavior. Fibers Polym..

[86] Herschel W.H., Bulkley R. (1926). Consistency measurements of rubber-benzene solutions. Kolloid-Zeitschrift.

[87] Casson N. (1959). A flow equation for pigment-oil suspensions of the printing ink type. Rheology of Disperse Systems.

[88] Li S.P., Zhao G., Chen H.Y. (2005). The Relationship between Steady Shear Viscosity and Complex Viscosity. J. Dispersion Sci. Technol..

[89] Beć K.B., Grabska J., Huck C.W. (2020). Near-infrared spectroscopy in bio-applications. Molecules.

[90] Bauer A.W., Kirby W.M., Sherris J.C., Turck M. (1966). Antibiotic susceptibility testing by a standardized single disk method. Am. J. Clin. Pathol..

